# Association between Social Activities and Cognitive Function among the Elderly in China: A Cross-Sectional Study

**DOI:** 10.3390/ijerph15020231

**Published:** 2018-01-30

**Authors:** Chang Fu, Zhen Li, Zongfu Mao

**Affiliations:** 1 Department of Social Medicine and Health Management, School of Health Sciences, Wuhan University, No.115 Donghu Road, Wuhan 430071, China; 2015103050006@whu.edu.cn; 2 Global Health Institute, Wuhan University, 115 Donghu Road, Wuhan 430071, China; 2015203050024@whu.edu.cn

**Keywords:** social activities, cognitive function, gender difference, elderly

## Abstract

Participation in social activities is one of important factors for older adults’ health. The present study aims to examine the cross-sectional association between social activities and cognitive function among Chinese elderly. A total of 8966 individuals aged 60 and older from the 2015 China Health and Retirement Longitudinal Study were obtained for this study. Telephone interviews of cognitive status, episodic memory, and visuospatial abilities were assessed by questionnaire. We used the sum of all three of the above measures to represent the respondent’s cognitive status as a whole. Types and frequencies of participation in social groups were used to measure social activities. Multiple linear regression analysis was used to explore the relationship between social activities and cognitive function. After adjustment for demographics, smoking, drinking, depression, hypertension, diabetes, basic activities of daily living, instrumental activities of daily living, and self-rated health, multiple linear regression analysis revealed that interaction with friends, participating in hobby groups, and sports groups were associated with better cognitive function among both men and women (*p* < 0.05); doing volunteer work was associated with better cognitive function among women but not among men (*p* < 0.05). These findings suggest that there is a cross-sectional association between participation in social activities and cognitive function among Chinese elderly. Longitudinal studies are needed to examine the effects of social activities on cognitive function.

## 1. Introduction

According to the World Alzheimer report, over 46 million people were living with dementia worldwide in 2015, and this number is estimated to reach 131.5 million by 2050 [1]. Cognitive decline is the defining feature of Alzheimer’s disease (AD) and other dementias. It significantly increases the risk of functional dependence and poor quality of life in the elderly [2]. Cognitive decline may begin after midlife, but most often occurs at higher ages [3]. The elderly people who suffer from cognitive decline are more likely to experience few activities of daily living, and demand continuous care from their families and society. Therefore, the cognitive decline of elderly can increase the burden of family members and social insurance funds. The total estimated worldwide cost of dementia is US $818 billion, and it will reach a trillion dollars by 2018 [1]. Thus, to prevent or delay cognitive decline of the elderly, an effective population-based strategy needs to be established. Previous studies have shown that cognitive decline is associated with socio-demographic factors [4], life habits [5], chronic diseases [4], and social networks [6].

Participation is one of the three pillars of a World Health Organization (WHO) policy framework for an active aging society [7]. Encouraging the elderly to participate in social activities can provide positive value to relieve the negative impact of the aging population, such as promoting the health of the elderly and reducing the burden on social insurance and family [8]. The role of social activities in the health of older adults is increasing in today’s aging society. Previous studies have showed that participation in social activities is one of the key determinants of older adults’ health outcomes. A study conducted in Japan indicated that participation in social activities was associated with independent instrumental activities of daily living (IADL) among community-based elderly [9]. A study from Korea reported that a lack of social activities is significantly related to an increased risk of depression [10]. Studies also showed that participation in social activities has beneficial effects on cognitive function among the elderly in developed countries. A study from the U.S. showed that participating in few social activities led to cognitive decline among Japanese-American men [11]. Another report from Spain indicated that social disengagement was one of the risk factors of cognitive decline among community-dwelling elderly [6]. 

However, there is little literature that deciphers the relationship between social activities and cognitive function among Chinese elderly. China has the largest elderly population in the world. The total population in China had reached 1.38 billion, in which adults aged 60 years and older accounted for 16.7%, by the end of 2016 [12]. In China, more than 10% of the elderly suffer from cognitive impairment [13], and there are more than 10 million elderly with AD [14]. Besides, due to the different social and cultural backgrounds, types of social activities in China may be different from other countries. In addition to the traditional Chinese social activities, such as playing Mahjong and practicing Qigong, square dance has become increasingly popular in China. Because it is not restricted by space and time, square dance is easily accepted by elderly people. To explore which forms of social activities (i.e., types and frequencies of social activities) are associated with cognitive function may improve understanding of how to achieve active aging in China. 

Previous studies have indicated that there is a gender difference with relation to impacts of participation in social activities on health outcomes like functional ability [9,15] and depression [16]. They found that elderly women may have more beneficial effects from social activities than men, because elderly men tend to have benefited more from their spouses’ support, while elderly women are more likely to obtain significant kind support from other people rather than their spouses [17]. In addition, frequency is an important indicator of measurement of social activities. Musick et al. indicated that both too little participation and too frequent participation in volunteer activities are associated with negative impacts on the health of elderly [18]. Takeuchi et al. found that frequent participation in sports groups have negative impacts on dental health among Japanese elderly [19]. However, few studies have assessed gender difference and frequency of participation in social groups when looking at the impacts of social activities on cognitive function among elderly. 

Hence, the aim of our study is to investigate the association between social activities and cognitive function, according to the types and frequency of participation in social groups, with regard to gender among Chinese elderly.

## 2. Materials and Methods

### 2.1. Study Population

The data of this study were from the 2015 China Health and Retirement Longitudinal Study (CHARLS). The CHARLS was conducted by the National School of Development (China Centre for Economic Research) of Peking University, which aimed to collect data on the family life and community conditions of individuals aged 45 and above, in order to analyze population issues, especially those related to population aging. Using a multistage sampling method, 21,096 individuals, from 12,400 households in 450 village-level units and 150 county-level units, participated in the survey. CHARLS includes information about the socio-demographic background, health status and physical function, healthcare and health insurance, and social activities of individuals. 

The object of this study is the elderly. Therefore, a sample of 9785 individuals aged 60 and older was selected. We deleted the subjects with no information about cognitive function or participation in social activities. The total missing subjects were 819. Finally, a sample of 8966 was enrolled into our study.

The data was obtained by applying to National School of Development (China Centre for Economic Research) of Peking University. The secondary analysis of data from CHARLS did not require ethics approval. 

### 2.2. Assessment of Cognitive Function 

Four dimensions of cognitive function were measured in our study, which included orientation, attention, episodic memory, and visuospatial abilities. Orientation and attention were assessed by ten items from the Telephone Interview of Cognitive Status (TICS-10), which each ranged from 0 to 10 [20]. TICS-10 included serial subtraction of 7 from 100 (up to five times), date (month, day, year), day of the week, and season of the year. Episodic memory was assessed by immediate and delayed word recall. Immediate recall refers to asking the subjects to immediately recall as many words as they could after interviewers read a list of 10 Chinese nouns. Delayed recall refers to asking the subjects to recall as many of the original words as possible after four to 10 min. The episodic memory score consisted of the average number of immediate and delayed word recalls, and ranged from 0 to 10 [21]. Visuospatial ability was assessed by figure drawing. Respondents were shown a picture and asked to draw a similar figure. Respondents who successfully drew the picture got a score of one, and those who failed to draw the picture got a score of zero [20]. All four of the dimensions of cognitive function were assessed by face-to-face interview. The overall cognition score represented respondents’ cognitive status was the sum score of the TICS-10, word recall, and figure drawing, which ranged from 0 to 21 [22]. Higher scores indicate better cognitive function.

### 2.3. Assessment of Social Activities 

In the CHARLS questionnaire, social activities were defined as the person’s involvement in activities, which included interaction with friends, participating in hobby groups (such as playing Mahjong, chess, cards or participating in other hobby groups), participating in sports groups (such as going to dancing, fitness, practicing Qigong, and so on), participating in community-related organizations, and doing voluntary work [23]. The respondents were asked whether or not they did any of these activities in the last month. If the answer was yes, then the respondents were asked to demonstrate the frequency of those activities in the last month. The answers were categorized as not regularly, almost every week, or almost daily.

### 2.4. Covariates

Demographic characteristics, lifestyle factors, health status, depression, basic activities of daily living (BADL), IADL, and chronic diseases were considered as confounder factors in this study. Demographic characteristics included age, marital status, educational level, and community type. Marital status was classified as married/cohabitating or divorced/separated/widowed/never married. Educational level was classified into 4 categories: no formal education, elementary school, middle school, high school and above. Community type was divided into city and village. Lifestyle factors included smoking and drinking. Information regarding cigarettes smoking and alcohol drinking was classified as current/past or never. Health status was assessed based on self-rated health. The respondents were asked to rate their health status on a five-point scale, including very poor, poor, fair, good, very good. Self-rated health was categorized as good (including very good and good), fair, or poor (including very poor and poor). Depression was assessed by the 10-item Center for Epidemiologic Studies Depression Scale (CES-D 10) [24], which has been validated among elderly respondents in China [25]. The answers of the CES-D 10 include 4 options: (1) rarely, (2) some days (1–2 days per week), (3) occasionally (3–4 days per week), (4) most of the time (5–7 days per week). The participants’ answers were coded as 0 (rarely) to 3 (most of the time) for the negative questions, and as 3 (rarely) to 0 (most of the time) for the positive questions [26]. Information on chronic diseases, including hypertension and diabetes mellitus, was identified by a community doctor or nurse. BADL were evaluated based on individuals’ basic abilities, which included eating, dressing, using the toilet, getting in and out of bed, defecating, and bathing [27]. IADL were evaluated based on individuals’ ability to do daily housework, make a telephone call, cook, take medicine, go shopping, and manage finances [26]. Each answer was divided into four responses, as follows: “can do it by myself”, “have some difficulties”, “need help” and “cannot do it”. The elderly who had any difficulty in any item were classified as having BADL/IADL functional decline. 

### 2.5. Statistics Analysis

Data was analyzed by using the Statistical Package for the Social Sciences (SPSS) version 20.0 (SPSS Inc., Chicago, IL, USA), with a significance level of 0.05. Chi-square tests and *t*-tests were used to explore the univariate relationships between age, marital status, community type, education, smoking, drinking, self-rated health, hypertension, diabetes mellitus, depression, BADL/IADL, social activities, and cognitive function by gender. Multiple linear regression analysis was used to examine the relationship between social activities and cognitive function. The multiple linear regression models adjusted for potential confounders (age, marital status, educational level, community type, cigarette smoking, alcohol drinking, depression, hypertension, diabetes, and BADL/IADL). We carried out multiple imputations, using chained equations to address missing data. The results are based on five multiple-imputed replicates. 

## 3. Results

### 3.1. Sample Characteristics

Table 1 showed the characteristics of the study population. Of the 8966 participants (mean age 68.1 years, SD = 6.6), 49.7% were men and 50.3% were women. Of the participants, 80.1% lived in villages, and 77.0% were married/cohabitating. Over half of the participants did not receive a formal education (55.6%), and 52.4% were reported as having a fair health status. More than half of participants never smoked or drank (56.2%, 67.4%, respectively). The mean depression score was 8.6 ± 6.6. Of the participants, 60.1% had no hypertension, 87.9% had no diabetes, and over 60% had completely normal BADL and IADL. The overall cognitive score was 9.2 ± 4.5 in the sample. As shown in Table 1, there was a significant difference between genders in marital status, educational level, smoking and drinking status, depression, hypertension and diabetes, BADL and IADL, self-rated health, and cognitive function.

### 3.2. Characteristics of Type and Frequency of Social Activities

Table 2 shows the characteristics of type and frequency of social activities. Of participants, 32.8% had interacted with friends in the last month, 18.6% had participated in hobby groups, 7.6% had participated in sports groups, 2.5% had participated in community-related organizations, and 13.9% had done volunteer work. Women were more likely to interact with friends and to participate in sports groups when compared with men. Men were more likely to participate in hobby groups, community-related organizations, and do volunteer work when compared with women. The frequency of each social activity had a significant difference between genders.

### 3.3. Cross-Sectional Relationship between Social Activities and Cognitive Function

Table 3 showed the cross-sectional relationship between types and frequencies of social activities and cognitive function. In model 1, interaction with friends, participating in hobby groups, participating in sports groups, participating in community-related organizations, and doing volunteer work were significantly associated with cognitive function among both men and women. In model 2, after adjusting for age, marital status, education, community type, smoking, and drinking, interaction with friends as well as participating in hobby groups and sports groups were significantly associated with better cognitive function among men; interaction with friends, participating in hobby groups, sports groups, and doing volunteer work were significantly associated with better cognitive function among women. In model 3, after adjusting for all covariates, interaction with friends (not regularly, B = 0.37, SE = 0.17, *p* = 0.026; almost every week, B = 0.62, SE = 0.21, *p* = 0.003; almost daily, B = 0.50, SE = 0.16, *p* = 0.002), participating in hobby groups (not regularly, B = 0.37, SE = 0.18, *p* = 0.043; almost every week, B = 1.05, SE = 0.21, *p* < 0.001; almost daily, B = 0.53, SE = 0.18, *p* = 0.004), and sports groups (almost daily, B = 0.60, SE = 0.24, *p* = 0.013) were significantly associated with better cognitive function among men; for women, interaction with friends (not regularly, B = 0.43, SE = 0.18, *p* = 0.019; almost daily, B = 0.29, SE = 0.15, *p* = 0.043), participating in hobby groups (not regularly, B = 1.24, SE = 0.27, *p* < 0.001; almost every week, B = 1.04, SE = 0.28, *p* < 0.001; almost daily, B = 0.63, SE = 0.25, *p* = 0.013), participating in sports groups (almost every week, B = 1.19, SE = 0.60, *p* = 0.048), and doing volunteer work (not regularly, B = 0.42, SE = 0.19, *p* = 0.026) were significantly associated with better cognitive function. The positive associations between participating in community-related organizations and cognitive function had been found in model 1 but not in model 3, which was explained by covariates. 

## 4. Discussion

To the best of our knowledge, this is the first study to assess the correlation between social activities and cognitive function among the Chinese elderly. In this cross-sectional analysis, our study found that the elderly who participated in social activities were significantly associated with better cognitive function. The results remained significant after adjusting for age, marital status, community type, education, smoking, drinking, self-rated health, hypertension, diabetes mellitus, depression, BADL, and IADL. Studies have indicated that participation in social activities has a positive impact on cognitive function among elderly. A previous longitudinal study in Korea for middle-aged and older individuals suggested that social activities may help preserve cognitive function [28]. Another study reported that more socially active older adults experience less cognitive decline [29]. Our results were consistent with these findings.

Base on the types of social activities, our results showed that participating in hobby groups, sports groups, and interaction with friends were positively associated with cognitive function in both genders, while doing voluntary work had a positive significantly association with cognitive function among women, not among men. Participation in community-related organizations had no significantly association with cognitive function among both genders. There are several possible explanations for the relationship between social activities and cognitive function. Participating in hobby groups (such as playing Mahjong, chess and cards, or other hobby groups) may enable individuals to have more complex and compound thinking, and mental training could help elderly prevent cognitive decline [30]. Participating in sports groups (such as dancing, fitness, practicing Qigong, or other sports groups) could promote physical activities for the elderly. A systematic review provides initial evidence that physical activities could benefit cognition in Chinese elderly [31]. Thus, participating in sports groups may help elderly maintain normal cognitive function. Interaction with friends could maintain or expand the social network of the elderly. Studies have indicated that extensive social network seems to protect against dementia [32,33]. Individuals with an expanded social network have more chances to access various forms of material resources or health-related information [34]. This may have a positive impact on health-related behaviors, which may protect people from cognitive decline. In addition, contacts with friends may provide a greater sense of purpose and emotional validation, which has direct neurohormonal benefits [35]. Our findings suggested that communities should create good conditions for social activities of the elderly, through establishing hobby activity rooms and constructing sport groups. Moreover, the elderly should be encouraged to interact with friends to maintain their social networks, so as to keep healthy cognitive function. 

The results of previous studies showed that doing volunteer work could improve cognitive function, through expanding older adults’ social networks and acquiring new learning [36,37]. However, we found that there was a significant association between doing volunteer work and cognitive function among women, but not among men. A possible explanation for this phenomenon was that women have a wider range of emotional support sources, and easily make close friends from their networks [17]. Thus, women received more beneficial effects on health outcomes from various types of social activities than men [16,38]. Our study showed that men were more likely to do volunteer work when compared with women. In traditional Chinese culture, men usually seek meaning and identity by being valued in their work unit, while women pay more attention to the internal family. After retirement, men’s social networks may shrink faster than women’s. Thus, elderly men are more likely to achieve a sense of self-esteem and feel rewarded through expanding social networks by doing volunteer work. Therefore, our study suggested that the government should improve the information networks among members and volunteer organizations, which may help improve cognitive function in elderly participants in China, especially for elderly men.

In addition, previous studies also indicated that social activities have not only helpful effects on health, but also negative ones [8,19]. Participation in social groups may have the adverse aspect of social relationships, such as personal conflict [34]. These adverse sides of participation in social activities lead to potential risk for psychological stress [8], which has negative effects on individuals’ health [39]. Kawachi et al. [40] pointed out that frequent participation in social activities may bring about psychological distress for women and adversely impact on their health. These negative effects of social activities had also been found in this study. For women, our results found that there was no significant association of both frequent volunteer work and participation in sports groups with cognitive function, but there was a positive significant association of comparatively infrequent volunteer work and participating in sports groups with cognitive function. This is consistent with Kawachi’s argument [40]. For both men and women, participation in community-related organizations had no significant association with cognitive function. This is consistent with the view that participation in community-related associations might include obligatory activities, which have the negative aspects of social networks [36]. The negative aspects of social networks might offset the helpful effects from participating in community-related associations. The limitations in this study are as follows. First, this cross-sectional survey study did not investigate the causal relationships between social activities and cognitive function. Since elderly with poor cognitive function may restrain themselves from participation in social groups, longitudinal studies are needed to examine the effects of social activities on cognitive function. Second, the survey depended on self-reports, which involved a risk of recall bias due to false or inaccurate responses from the participants. Third, the CHARLS could not assess the quality of interactions in social activities. The quality of interaction is an important indicator of participation in social activities [41]. The negative social experiences in social activities impact health differently than positive experiences [42], and women may be more sensitive to the quality of social relations than men [43]. The quality of interaction in social activities should be considered in the future studies.

## 5. Conclusions 

In conclusion, our study showed that interaction with friends, participating in hobby groups, and participating in sports groups were associated with better cognitive function among both elderly men and women in China, while doing volunteer work was only positively associated with cognitive function among women. The present study indicated that the possibility that participation in social activities is protective of cognitive function. Since the present cross-sectional design could not exclude the possibility that elderly people with better cognitive function tend to do more social activities than elderly people with poor cognitive function, longitudinal studies are suggested to examine the causal relationship between social activities and cognitive function. 

## Figures and Tables

**Table 1:** Characteristics of the study population.

Variables	Total (*n* = 8966)	Men (*n* = 4457)		Women (*n* = 4509)	*p* Value
Age, years, mean ± SD	68.1 ± 6.6	68.2 ± 6.5		68.0 ± 6.6	0.121
Marital status, (%)					<0.001
Married/cohabitating	77.0	82.9		71.1	
Divorce/separated/widowed/never married	23.0	17.1		28.9	
Community type, (%)					0.348
Village	80.1	80.5		79.7	
City	19.9	19.5		20.3	
Educational level, (%)					<0.001
No formal education	55.6	40.4		70.7	
Elementary school	23.0	29.8		16.3	
Middle school	13.9	19.0		8.9	
High school or above	7.5	10.9		4.1	
Smoking, (%)					<0.001
Current/Past	43.8	77.9		10.1	
Never	56.2	22.1		89.9	
Alcohol consumption, (%)					<0.001
Current/Past	32.6	51.4		14.1	
Never	67.4	48.6		85.9	
Depression, mean ± SD	8.6 ± 6.6	7.4 ± 6.0		9.9 ± 6.9	<0.001
Hypertension, (%)					<0.001
Yes	39.9	37.3		42.5	
No	60.1	62.7		57.5	
Diabetes, (%)					<0.001
Yes	12.1	10.4		13.9	
No	87.9	89.6		86.1	
Self-rated health, (%)					<0.001
Good	21.5	23.8		19.2	
Fair	52.4	52.6		52.2	
Poor	26.1	23.5		28.6	
BADL, (%)					0.012
Completely normal	66.2	67.8		64.9	
Functional decline	33.8	32.2		35.1	
IADL, (%)					<0.001
Completely normal	61.8	70.8		53.0	
Functional decline	38.2	29.2		47.0	
Cognitive measure					
TICS (0–10), mean ± SD	6.0 ± 3.0	6.9 ± 2.7		5.1 ± 3.1	<0.001
Draw a figure (0/1), mean ± SD	0.5 ± 0.5	0.7 ± 0.5		0.4 ± 0.5	<0.001
Words recall (0–10), mean ± SD	2.7 ± 1.8	2.9 ± 1.8		2.6 ± 1.9	<0.001
Overall cognition (0–21), mean ± SD	9.2 ± 4.5	10.4 ± 4.0		8.2 ± 4.6	<0.001

Note: SD, standard deviation; BADL, basic activities of daily living; IADL, instrumental activities of daily living; TISC, telephone interview of cognitive status. Percentages may not add up to 100% due to rounding.

**Table 2:** Characteristics of type and frequency of social activities.

Type and Frequency of Social Activities	Total (*n* = 8966)	Men (*n* = 4457)	Women (*n* = 4509)	*p* Value
Interaction with friends, (%)				<0.001
No participant	67.2	69.1	65.3	
Not regularly	10.9	11.4	10.3	
Almost every week	6.6	7.0	6.2	
Almost daily	15.3	12.4	18.2	
Hobby groups, (%)				<0.001
No participant	81.4	76.4	86.4	
Not regularly	6.5	8.5	4.5	
Almost every week	5.2	6.5	4.0	
Almost daily	6.9	8.6	5.2	
Sports groups, (%)				<0.001
No participant	92.4	93.9	90.9	
Not regularly	1.3	0.7	1.8	
Almost every week	0.7	0.6	0.9	
Almost daily	5.6	4.8	6.4	
Community-related organization, (%)				<0.001
No participant	97.5	97.1	97.9	
Not regularly	1.6	2.1	1.1	
Almost every week	0.6	0.7	0.6	
Almost daily	0.3	0.2	0.5	
Volunteer, (%)				0.001
No participant	86.1	84.9	87.3	
Not regularly	10.3	11.3	9.4	
Almost every week	2.0	2.4	1.6	
Almost daily	1.6	1.5	1.7	

Percentages may not add up to 100% due to rounding.

**Table 3:** Multiple linear regression model testing the association between social activities and cognitive score.

Gender	Social Activities	Model 1			Model 2		Model 3	
		B (SE)	*p*	B (SE)		*p*	B (SE)	*p*
Men	Interaction with friends (ref. no participant)							
	Not regularly	0.89 (0.19)	<0.001	0.39 (0.17)		0.020	0.37 (0.17)	0.026
	Almost every week	1.27 (0.24)	<0.001	0.65 (0.21)		0.002	0.62 (0.21)	0.003
	Almost daily	0.54 (0.19)	0.004	0.55 (0.16)		0.001	0.50 (0.16)	0.002
	Hobby groups (ref. no participant)							
	Not regularly	0.88 (0.22)	<0.001	0.49 (0.19)		0.010	0.37 (0.18)	0.043
	Almost every week	1.86 (0.24)	<0.001	1.25 (0.21)		<0.001	1.05 (0.21)	<0.001
	Almost daily	1.08 (0.21)	<0.001	0.76 (0.19)		<0.001	0.53 (0.18)	0.004
	Sports groups (ref. no participant)							
	Not regularly	0.33 (0.70)	0.637	−0.49 (0.61)		0.428	−0.62 (0.60)	0.295
	Almost every week	1.48 (0.76)	0.052	0.09 (0.67)		0.897	−0.06 (0.65)	0.931
	Almost daily	1.79 (0.28)	<0.001	0.75 (0.25)		0.003	0.60 (0.24)	0.013
	Community-related organization (ref. no participant)							
	Not regularly	0.94 (0.42)	0.025	0.25 (0.37)		0.504	0.18 (0.36)	0.615
	Almost every week	1.13 (0.74)	0.127	0.54 (0.64)		0.406	0.47 (0.63)	0.458
	Almost daily	2.34 (1.31)	0.074	1.60 (1.15)		0.164	1.22 (1.12)	0.275
	Volunteer (ref. no participant)							
	Not regularly	0.42 (0.19)	0.030	0.15 (0.17)		0.384	0.13 (0.16)	0.416
	Almost every week	0.27 (0.40)	0.495	0.04 (0.34)		0.916	0.17 (0.33)	0.623
	Almost daily	0.32 (0.49)	0.513	−0.18 (0.43)		0.676	−0.19 (0.42)	0.648
Women	Interaction with friends (ref. no participant)							
	Not regularly	0.52 (0.22)	0.021	0.45 (0.19)		0.017	0.43 (0.18)	0.019
	Almost every week	0.89 (0.28)	0.001	0.56 (0.24)		0.019	0.44 (0.23)	0.057
	Almost daily	−0.05 (1.78)	0.760	0.33 (0.15)		0.029	0.29 (0.15)	0.043
	Hobby groups (ref. no participant)							
	Not regularly	2.93 (0.32)	<0.001	1.58 (0.27)		<0.001	1.24 (0.27)	<0.001
	Almost every week	2.45 (0.34)	<0.001	1.23 (0.29)		<0.001	1.04 (0.28)	<0.001
	Almost daily	1.95 (0.30)	<0.001	0.95 (0.26)		<0.001	0.63 (0.25)	0.013
	Sports groups (ref. no participant)							
	Not regularly	1.43 (0.50)	0.004	0.38 (0.42)		0.368	0.44 (0.41)	0.282
	Almost every week	2.58 (0.73)	<0.001	1.31 (0.62)		0.035	1.19 (0.60)	0.048
	Almost daily	2.29 (0.28)	<0.001	0.47 (0.24)		0.048	0.27 (0.23)	0.238
	Community-related organization (ref. no participant)							
	Not regularly	1.53 (0.66)	0.019	0.40 (0.56)		0.474	0.33 (0.54)	0.545
	Almost every week	1.32 (0.90)	0.141	0.68 (0.76)		0.373	0.62 (0.74)	0.400
	Almost daily	2.84 (0.96)	0.003	0.73 (0.81)		0.369	0.56 (0.79)	0.481
	Volunteer (ref. no participant)							
	Not regularly	0.88 (0.23)	<0.001	0.48 (0.20)		0.013	0.42 (0.19)	0.026
	Almost every week	0.52 (0.53)	0.333	−0.13 (0.45)		0.778	−0.04 (0.43)	0.928
	Almost daily	1.25 (0.51)	0.015	0.88 (0.44)		0.044	0.78 (0.42)	0.063

Note: B, Coefficient; SE, Standard error, Model 1: crude model. Model 2: adjusted for age, marital status, education, community type, smoking and drinking. Model 3: adjusted for age, marital status, education, community type, smoking, drinking, hypertension, diabetes, depression, self-rated health, BADL and IADL.
